# The efficacy of adding oral sodium cromoglycate to stable treatment for controlling bullous pemphigoid-related pruritus: A retrospective study

**DOI:** 10.3389/fmed.2022.1051804

**Published:** 2022-12-08

**Authors:** Noy Keller Rosenthal, Darby Boucher, Dedee F. Murrell

**Affiliations:** ^1^Department of Dermatology, Faculty of Medicine, St. George Hospital, University of New South Wales, Sydney, NSW, Australia; ^2^The George Institute for Global Health, Sydney, NSW, Australia

**Keywords:** bullous pemphigoid (BP), itch, pruritus, sodium cromoglycate, cromolyn disodium, itch therapy

## Abstract

**Introduction:**

Bullous pemphigoid (BP) is the most common autoimmune subepidermal blistering disease which mainly affects the elderly. It manifests as severe pruritus, urticarial plaques, and tense bullae and is associated with significant mortality. Therapy options for itch in BP patients are limited, mainly because the pathogenesis of itch in BP remains unclear. Sodium cromoglycate was commonly used in the past as an inhaled drug for the management of bronchial asthma and as an oral treatment for children with urticaria pigmentosa. In this study we sought to assess its efficacy in reducing BP associated itch.

**Objective:**

Assessing the efficacy of oral sodium cromoglycate in reducing BP-related pruritus after stabilization of disease activity.

**Methods:**

We retrospectively reviewed the medical records of patients with a confirmed diagnosis of BP who were treated with sodium cromoglycate. Patient reported outcome measures (PROM) including: BPDAI pruritus, ABQOL and TABQOL, and BPDAI activity score were compared at two points in time: before commencing treatment with sodium cromoglycate or before commencing maximal dose of this treatment, and at least 4 weeks after treatment commencement.

**Results:**

A total of 21 patients met the inclusion criteria. After at least 4 weeks of treatment with oral sodium cromoglycate BPDAI pruritus, ABQOL and TABQOL scores were statistically significantly decreased compared to the scores prior to treatment commencement, *P* < 0.000, *P* < 0.008, and *P* < 0.004, respectively.

**Discussion:**

Oral treatment with sodium cromoglycate for the management of pruritus in BP patients may be beneficial, however, further prospective studies are required to better assess its efficacy.

## Introduction

Bullous pemphigoid (BP) is the most common autoimmune subepidermal blistering skin disease and is most prevalent in the elderly (1, 2). BP is characterized by the presence of autoantibodies against distinct structural components of the dermal–epidermal junction: BP180 antigen (BP180 or BPAG2) and BP230 antigen (BP230 or BPAG1). The latter proteins link the cytoskeleton of the basal keratinocytes to the extracellular matrix of the dermis, promoting dermal-epidermal cohesion. Binding of pemphigoid autoantibodies leads to the separation of the epidermis and dermis by a complex inflammatory process, involving complement fixation, that is yet to be fully understood (3–5).

The main symptom of BP is localized, or more frequently, generalized tense blister formation which is associated with significant morbidity and mortality (1, 6). Urticarial plaques often precede the development of bullae, but can also constitute the only cutaneous manifestation, in an increasingly recognized subtype of BP.

Another major symptom of BP is severe itch which remarkably impacts the patients’ quality of life. In some patients, severe itch can be the only manifestation of the disease (2, 3).

Controlling pruritus is a significant challenge in the management of BP (7, 8). Current therapeutic paradigms for BP are aimed at immune regulation and include corticosteroids and immunosuppressants (9), which are partially competent in alleviating BP-associated itch, but known to have major adverse effects, especially in long term use and in frail, elderly patients. Since little is known about the underlying pathophysiology of itch in BP, the therapeutic alternatives are limited.

Recent studies have shown a correlation between multiple itch mediators, including eosinophils, neurokinin 1R (NK1R), substance P (SP), interleukin (IL) 31 receptor A and oncostatin M receptor-β, IL-13, periostin, and basophils and itch severity of patients with BP (7, 8).

In the last decade, NK1R and its ligand SP have been marked as novel therapeutic targets for the treatment of chronic pruritus (10). NK1R is expressed by sensory nerve fibers, by cells in the central nervous system, and also by various types of cells, including keratinocytes, mast cells, and eosinophils (11). SP stimulates NK1R-expressing cells, resulting in the release of additional itch mediators (12).

Sodium cromoglycate is a synthetic bischromone derivative that was commonly used in the past as an inhaled drug in the prophylaxis and adjunctive management of bronchial asthma, and as an oral treatment in children with urticaria pigmentosa. It is believed to act by stabilization of mast cell membranes, thereby inhibiting the release of pharmacological mediators of anaphylaxis when the cells are triggered in a selective manner (13, 14). It is poorly absorbed through the gut and is taken daily for about 4 weeks to reach steady state (15). Sodium cromoglycate efficacy in reducing pruritus was compared against placebo in two studies so far. A significant reduction in pruritus intensity was observed in the cromolyn group, despite the patients having difficult-to-treat chronic kidney disease-related pruritus (16, 17).

In this study we retrospectively assessed sodium cromoglycate efficacy in reducing BP-associated pruritus in patients who were stable on other therapies apart for itch.

## Objective

Assessing the efficacy of oral sodium cromoglycate in reducing BP-related pruritus after stabilization of disease activity.

## Methods

### Inclusion

This was an observational, retrospective, single-center study from an academic blistering disease center in Sydney, Australia. We reviewed the clinical records of all patients with the diagnosis of BP. Patients with a confirmed diagnosis of BP and a history of treatment with oral sodium cromoglycate were included in the study. A confirmed diagnosis include a consistent clinical diagnosis and a lesional biopsy histology consistent with BP and positive DIF on perilesional skin and positive IIF on human BMZ-split skin or positive BP180/BP230 ELISA and/or diagnosis with BIOCHIP (18). Patients had to have stable disease activity but active pruritus.

### Patient reported outcome measures

BPDAI pruritus, ABQOL and TABQOL and BPDAI activity score are collected routinely at these clinics and were compared at two points in time for each patient: before commencing treatment with sodium cromoglycate or before commencing maximal dose of this treatment, and at least 4 weeks after treatment commencement.

### Exclusion

Patients were excluded from the study if data was partial or insufficient or if sodium cromoglycate was commenced in conjunction with another immunosuppressive drug/in conjunction with commencement of corticosteroids. When the patient’s treatment scheme included long standing oral corticosteroids, they were included in the study only if they were in the process of tapering down oral corticosteroids, or on a stable dose for more than 4 weeks (recalcitrant BP). With regard to other systemic treatments, they had to be on a stable dose for at least 4 weeks to meet inclusion criteria. These were defined as exclusion criteria in order to minimize the confounding effect of these treatments on the efficacy of sodium cromoglycate in controlling pruritus.

### Statistics

In order to evaluate whether there was a change to the patients’ PROMs as a result of treatment with oral sodium cromoglycate, a Wilcoxon signed rank test was performed using SPSS version 26.

## Results

A total of 43 BP patients’ records were screened. 10 patients were excluded due to insufficient data, 6 were excluded due to the use of inhaled sodium cromoglycate, 5 were excluded due to a change in concomitant therapy with the commencement of sodium cromoglycate and one patient did not take the prescribed sodium cromoglycate.

In total, 21 patients were included in the study (10 males, 11 females). The patients presented at the clinic between 2014 and 2022. The overall mean age was 72.1 (SD 9.36). Further information regarding the patients’ characteristics is presented in Table 1. A Wilcoxon signed rank test showed that after at least 4 weeks of treatment with oral sodium cromoglycate BPDAI pruritus and ABQOL scores were statistically significantly decreased compared to the scores prior to treatment commencement, *Z* = 24.824, *P* < 0.000 and *Z* = 22.94, *P* < 0.008, respectively. TABQOL before and after at least 4 weeks of treatment with sodium cromoglycate was also statistically significantly reduced *Z* = 24.832, *P* < 0.004. Patients’ scores are provided in Table 2.

**TABLE 1:** Patients characteristics: demographics, duration of disease, and sodium cromoglycate dosage.

Patient no. characteristics	1	2	3	4	5	6	7	8	9	10	11	12	13	14	15	16	17	18	19	20	21
Gender	M	F	M	F	F	F	M	M	F	M	F	M	M	F	F	F	M	M	F	F	M
Age	74	72	60	65	67	54	70	80	78	71	61	82	86	62	59	69	79	78	81	83	6
Duration of BP (Months)	48	24	24	12	5	3	96	4	12	6	9	12	5	5	156	6	6	20	12	4	21
Daily dose of sodium cromoglycate*	0.2	1	1	1	0.2	1	3	1	1	1	2	1	1	1	1	1	1	1	1	1	2

*Number of 100 mg tablets.

**TABLE 2:** Bullous pemphigoid (BP) patient reported outcome measures (PROMs) scores pre and at least 4 weeks post-commencement of sodium cromoglycate.

Patient no. score	1	2	3	4	5	6	7	8	9	10	11	12	13	14	15	16	17	18	19	20	21
BPDAI pruritus pre	3	14	15	19	6	10	3	9	15	6	3	0	21	18.5	21	24	24	12	18	0	7
BPDAI pruritus post	0	7.5	11	6	5	12	1	4	9	5	0	0	0	11	19	3	9	7	5	0	3
ABQOL pre	6	32	22	20	10	17	17	30	29	6	9	4	18	23	36	14	14	22	11	18	5
ABQOL post	0	12	22	1	11	23	14	16	12	5	3	0	30	12	36	7	0	3	13	18	3
TABQOL pre	9	28	27	18	9	31	14	27	38	9	15	1	14	12	42	16	12	31	26	28	13
TABQOL post	0	12	23	0	4	31	17	20	25	7	8	1	40	10	40	11	0	10	16	20	1

Sodium cromoglycate doses varied between 20 mg daily and 100 mg three times daily. The majority of patients received 100 mg daily (*n* = 16).

A total of 16 patients were on concomitant therapy with topical corticosteroids, 13 were treated with tetracyclines, and 8 of them received nicotinamide. 4 patients were getting monthly IVIG for at least 2 months and 10 were on concomitant therapy of oral corticosteroids but were either on a stable long-term dose (*n* = 1) or in the process of tapering down corticosteroids during the period of time that was reviewed (*n* = 9). When comparing BPDAI pruritus scores pre and post-treatment with sodium cromoglycate within the group of patients who did not receive oral corticosteroids, it is statistically significantly decreased, *Z* = 9.798, *P* < 0.05, as well as within the group of patients who received oral corticosteroids, *Z* = 8.434, *P* < 0.021. Further details on patients’ combination therapy is provided in Table 3.

**TABLE 3:** Bullous pemphigoid (BP) patients combination therapies at commencement of sodium cromoglycate.

Patient no. treatment	1	2	3	4	5	6	7	8	9	10	11	12	13	14	15	16	17	18	19	20	21
IVIG		+	+				+											+			
Oral corticosteroids			+		+	+		+		+				+		+	+		+	+	
MTX		+					+														
MMF								+							+						
Tetracycline	+	+	+	+	+	+				+	+	+	+				+	+	+		
Nicotinamide		+	+	+	+					+							+	+	+		
Topical corticosteroids	+	+				+		+	+	+	+	+	+	+	+	+	+	+	+		+
Calcineurin inhibitors							+														

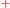
 In the process of tapering oral corticosteroids down.

+ Stable dose.

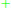
 IVIG started more than 4 months prior to sodium cromoglycate.

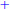
 IVIG started 2 months prior to sodium cromoglycate.

## Limitations

The limitations of this study include its observational retrospective design, the sample size, and the short follow-up time.

## Discussion

Bullous pemphigoid is the most common type of autoimmune subepidermal autoimmune bullous diseases. It typically manifests as a generalized pruritic bullous skin eruption, and is potentially associated with significant morbidity (1, 6). In up to 20% of affected patients, bullae may be completely absent, and only excoriations, prurigo-like lesions, eczematous lesions, urticated lesions, and/or infiltrated plaques are observed (19). A shared major symptom to all subtypes of BP is intense pruritus which is a burdensome condition that substantially affects patients’ quality of life. While BP is considered to be a rare disease, its incidence has been reported to increase from 1.9- to 4.3-fold in the past two decades in several countries across the world (1).

Despite the increasing incidence and awareness of it, evidence about effective treatment for BP-related pruritus is sparse. Perhaps a putative explanation is that underlying mechanisms of BP-related pruritus are still mostly unknown (7, 8).

Sodium cromoglycate is considered a safe drug with a limited profile of side effects (16) which is believed to act as a mast cell stabilizer. Nicotinamide, a potent stabilizer of mast cells and leukocytes, was also prescribed to eight of our patients, however, in a previous study it was not found to be more effective than placebo in reducing uremic pruritus (20, 21).

Our study results suggest that oral treatment with sodium cromoglycate for the management of pruritus in BP patients may be beneficial. Our cohort of patients received varying doses of sodium cromoglycate with the vast majority responding to 100 mg daily, hence, we recommend this dosing as initial dose. The difference between patients could be related to absorption differences among the patients as well as to the severity of primary symptoms and should be further investigated by a randomized controlled trial. We were able to demonstrate statistically significant improvements in patients’ pruritus and quality of life scores, however, further clinical trials with a prospective design are still needed for the assessment of sodium cromoglycate, or other mast cell stabilizers efficacy in BP-related itch.

## Data availability statement

The raw data supporting the conclusions of this article will be made available by the authors, without undue reservation.

## Author contributions

NK have made a substantial contribution to the design, analysis and interpretation of data, and drafting of manuscript. DB have made a substantial contribution to the data collection. DM have made a substantial contribution to the design of the manuscript, revised it critically for important intellectual content, and agreed to be accountable for all aspects of the work ensuring that questions related to the accuracy or integrity of any part of the work were appropriately investigated and resolved. All authors contributed to the article and approved the submitted version.
